# Association between household environment and the risk of depression and anxiety among women in Bangladesh: analysis of the Bangladesh Demographic and Health Survey (2022)

**DOI:** 10.1016/j.lansea.2026.100840

**Published:** 2026-08-17

**Authors:** Md Mostaured Ali Khan, Kaniz Fatima, Md Niamul Islam Sium, Md Arif Billah, Md Golam Mustagir, Sumyta Rahman, Md Nuruzzaman Khan, Golam M. Khandaker

**Affiliations:** a MRC Integrative Epidemiology Unit, Bristol Medical School, University of Bristol, Bristol, UK; b Centre for Academic Mental Health, Department of Population Health Sciences, Bristol Medical School, University of Bristol, Bristol, UK; c Maternal and Child Health Division (MCHD), icddr,b, 68 Shaheed Tajuddin Ahmed Sarani, Mohakhali, Dhaka 1212, Bangladesh; d Nutrition Research Division (NRD), icddr,b, 68 Shaheed Tajuddin Ahmed Sarani, Mohakhali, Dhaka 1212, Bangladesh; e Data Science, Department of Mathematical Sciences, The University of Texas at El Paso, 500 W. University Ave. El Paso, TX, 79968, USA; f Department of Public Health, School of Psychology and Public Health, La Trobe University, Bundoora, VIC, 3086, Australia; g Health Systems and Population Studies Division (HSPSD), icddr,b, 68 Shaheed Tajuddin Ahmed Sarani, Mohakhali, Dhaka 1212, Bangladesh; h Projahnmo Research Foundation, Banani, Dhaka 1213, Bangladesh; i Department of Public Health, North South University, Bashundhara, Dhaka 1229, Bangladesh; j Nossal Institute for Global Health, Melbourne School of Population and Global Health, The University of Melbourne, Grattan Street, Parkville, VIC, 3010, Australia; k Department of Population Science, Jatiya Kabi Kazi Nazrul Islam University, Namapara, Mymensingh 2220, Bangladesh; l NIHR Bristol Biomedical Research Centre and NIHR Bristol Clinical Research Facility, University Hospitals Bristol and Weston NHS Foundation Trust, Bristol, UK; m Avon and Wiltshire Mental Health Partnership NHS Trust, Bristol, UK

**Keywords:** Mental health, Depression, Generalised anxiety disorder, Housing condition, Household environment, Women's health, Bangladesh

## Abstract

**Background:**

Depression and anxiety are common mental disorders globally but often overlooked in low- and middle-income countries (LMICs), with limited population-level evidence on the role of household environmental conditions (HECs), especially for women. This study examined associations of HECs with depression and anxiety among women in Bangladesh.

**Methods:**

This cross-sectional study analysed data from ever-married women in the 2022 Bangladesh Demographic and Health Survey. Depression and anxiety symptoms were measured using validated PHQ-9 and GAD-7 scales. Exposures included seven HEC indicators—housing materials, overcrowding, household air pollution (HAP), water source and treatment, sanitation, and handwashing facilities—and a cumulative HEC score. Associations were assessed using multinomial logistic regression and reported as adjusted risk ratios (aRR) with 95% confidence intervals (95% CI), adjusting for age, education, employment, place and duration of residence, etc.

**Findings:**

Among 19,012 women, prevalence of probable depression and generalised anxiety disorder (GAD) was 5·0% and 4·4%, respectively. More than half of households had ≥3 poor HECs. Compared with women in households with ≤1 poor HEC, those with three (aRR: 1·30, 95% CI: 1·13–1·83) or ≥4 poor HECs (aRR: 1·31, 95% CI: 1·02–1·67) had higher risk of depression but not GAD. High HAP exposure was associated with both depression (aRR: 1·58, 95% CI: 1·01–2·45) and GAD (aRR: 1·61, 95% CI: 1·02–2·57). Overcrowding was associated with probable GAD (aRR: 1·32, 95% CI: 1·07–1·63), and poor sanitation with depression (aRR: 1·21, 95% CI: 1·05–1·39) only.

**Interpretation:**

Poor HECs are potential contributors to depression and anxiety among Bangladeshi women. Integrating environmental improvements, particularly reducing HAP, improving sanitation, and reducing household overcrowding, into mental health prevention strategies may complement existing efforts to reduce the burden of common mental disorders in Bangladesh and other similar LMICs.

**Funding:**

The work was supported by UKRI Medical Research Council grant (grant no: MC_UU_00032/6).

**Research in context**

**Evidence before this study**

Mental health problems are a growing concern in low- and middle-income countries (LMICs), where populations are highly exposed to household-level environmental risks, such as poor-quality housing, unsafe drinking water, inadequate sanitation, and indoor air pollution. These exposures can act as chronic stressors and contribute to adverse mental health outcomes. Women, especially in rural areas, who typically spend most of their time in household work are particularly vulnerable. Despite this, population-based evidence on the associations between household environmental conditions (HECs) and women's mental health in LMICs, including Bangladesh, remains scarce. We searched PubMed, Web of Science, and Google Scholar for studies published in English up to July 2025 that examined HECs and common mental disorders in LMICs. The search terms covered mental health outcomes, housing quality, overcrowding, household air pollution, water, sanitation, and hygiene, together with relevant synonyms and related terms. Existing evidence indicates that some HECs, such as household air pollution, water insecurity, and inadequate sanitation, are associated with depression and anxiety. However, existing studies typically focused on socio-demographic, psychosocial, or behavioural risk factors. Evidence on HECs is typically limited to a few indicators, and rarely considers cumulative or multidimensional exposures, particularly among women.

**Added value of this study**

We analysed BDHS (2022) survey data of women in Bangladesh to estimate the prevalence of depression and anxiety using validated screening instruments. We examined their associations with multiple HECs moving beyond conventional socioeconomic measures. We assessed both individual HEC indicators and a cumulative HEC score, and adjusting for socioeconomic factors to identify specific, potentially modifiable household-level environmental determinants. We further distinguish between clinically relevant and mild symptom profiles, revealing patterns not captured in earlier research.

**Implications of all the available evidence**

Taken together, the evidence suggests that HEC represent an under-recognised but actionable pathway influencing women's mental health in LMICs. Our findings emphasise on integrating improvements in housing quality, clean cooking facilities, and safer sanitation into mental health and public health strategies may yield additional benefits beyond traditional psychosocial interventions. Policies addressing household environments could complement existing mental health programmes and contribute to more holistic and preventive approaches to reducing the burden of depression and anxiety among women in Bangladesh.

## Introduction

Depression and anxiety are the most prevalent mental disorders worldwide, affecting an estimated 359 million and 332 million people, respectively.^1^^,^^2^ They substantially increase the risk of a range of chronic diseases,^3^^,^^4^ impaired functioning,^4^ suicidal behaviour,^4^ and poor maternal and child health as well.^4^ Consequently, these disorders are among the top ten leading causes of disability worldwide, accounts for approximately 42·5 and 56·3 million years of healthy life lost each year, respectively.^1^^,^^2^ They arises from a multifactorial and complex interplay of biological and psychosocial factors^5^, ^6^, ^7^ and their disease burden notably varies across regions worldwide.^1^^,^^2^ Low- and middle-income countries (LMICs) account for around 85% of the global burden of mental illness, yet mental health care remains under-resourced and often overlooked in LIMCs.^8^ Understanding context-specific risk factors is, therefore, crucial to reduce the burden and achieve 3·4 Sustainable Development Goal (SDG 3·4), which aims to promote mental health and well-being to reduce morbidity in overall population.^9^

In recent years, environmental and housing-related factors have emerged as important yet underexplored determinants of mental disorders.^10^ Household environmental conditions (HECs), such as access to safe water, sanitation and hygiene, housing materials, and household air pollution (HAP), etc., which defines the material and infrastructural features of the household and represent direct measures of an individual's immediate physical living environment.^10^^,^^11^ As such, HECs provide key information regarding the household environment above and beyond those reflected by conventional measures of socioeconomic status (SES), which primarily includes structural resources such as income, education or occupation.^10^, ^11^, ^12^ Globally, especially in LMICs, still 2·8 billion people lack safe sanitation, 1·6 billion lack safe drinking water at home, and 2·1 billion use solid fuels for cooking.^13^^,^^14^ Although a growing body of recent global evidence has linked some specific HECs, such as HAP, inadequate sanitation, drinking water source, etc., with depression and/or anxiety,^10^^,^^15^, ^16^, ^17^, ^18^ none of them considered all the indicators of HECs that may represent distinct and modifiable determinants of mental health.

Women's mental health remains under-researched in LMICs despite its being prioritised globally.^19^ They are disproportionately affected by depression and anxiety as these disorders are generally more common among women than men.^20^ Existing work on risk factors for depression and anxiety in LMICs, including Bangladesh, has largely focused on socio-demographic,^21^, ^22^, ^23^, ^24^, ^25^ psychosocial and behavioural risk factors,^26^^,^^27^ such as income,^22^^,^^23^^,^^28^ education,^22^^,^^25^ adverse life experiences,^26^^,^^27^ food insecurity,^28^^,^^29^ physical inactivity,^29^ etc. Many women in Bangladesh continue to spend most of their time at home in domestic roles,^14^ and, as a result, they are highly exposed to poor HECs. However, to our knowledge, no studies have examined the relationship of HECs with depression and anxiety in a representative sample of women from Bangladesh or any LMICs. Such work could be useful for informing the development of targeted household-level health interventions to improve women's mental health in Bangladesh and similar LMICs.

This study aimed to examine the association between HEC indicators and the risk of depression and anxiety in a population-representative sample of reproductive age women in Bangladesh. In addition to testing the effects of specific HECs we examined the effect of cumulative poor HEC burden, and whether these relationships could be explained by traditional markers of SES such as education, employment, etc.

## Methods

**Fig. 1:**
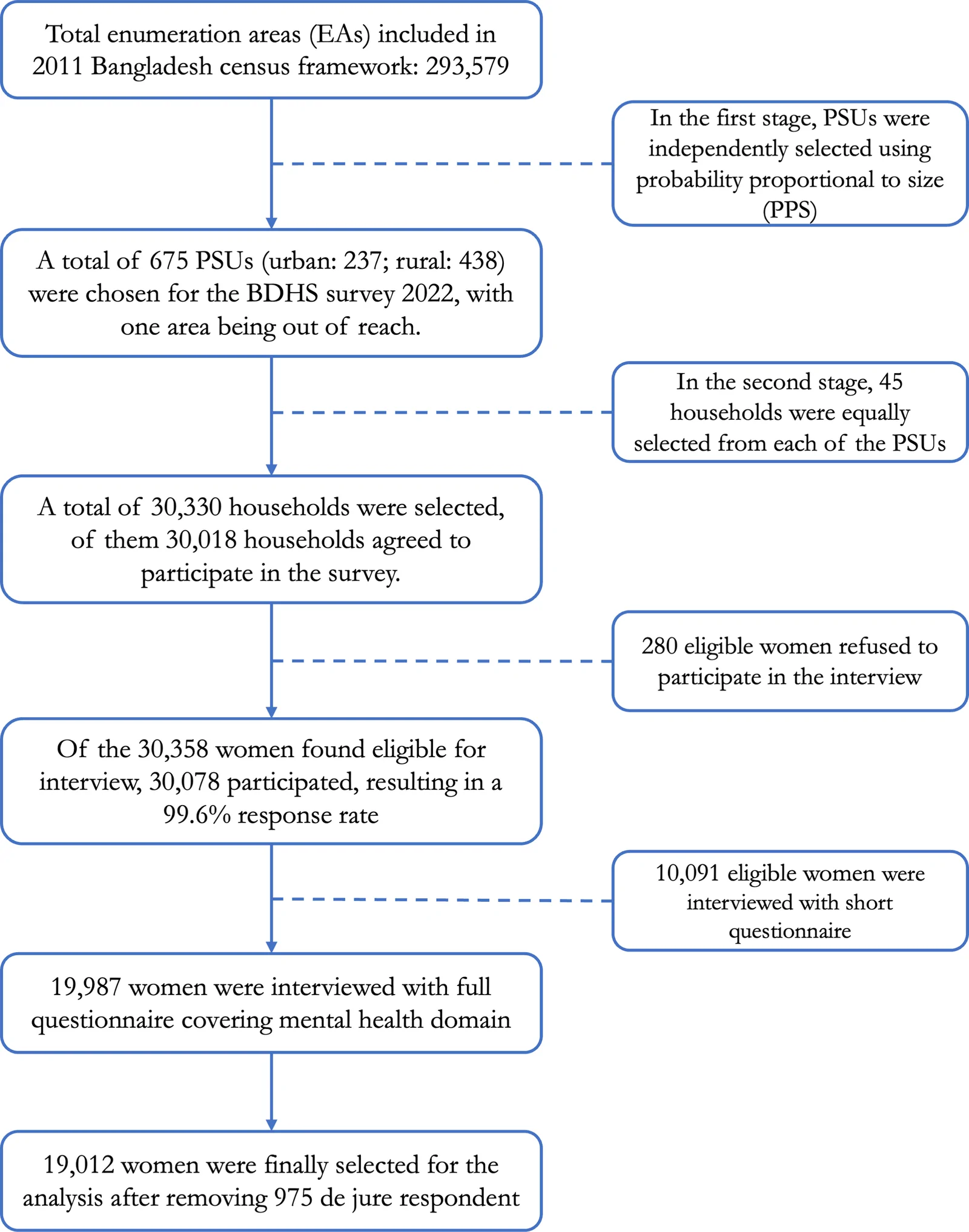
Flow chart of the sampling procedure and study participant selection.

This cross-sectional study analysed data from the 9th round of nationally representative 2022 Bangladesh Demographic and Health Survey (BDHS). The National Institute of Population Research and Training (NIPORT) conducts this cross-sectional survey in every three to four years of interval under the Ministry of Health and Family Welfare (MoHFW). The BDHS 2022 employed a two-stage stratified random sampling method to select the eligible households. In the first stage of sampling, 675 primary sampling units (PSUs) were randomly chosen using the probability proportional to size (PPS), consisting of urban and rural areas. These PSUs were chosen from a complete list of enumeration areas (EAs) produced by the Bangladesh Bureau of Statistics (BBS) for National Population Census in 2011. Data were collected from all selected PSUs expect one, which was unreachable. In the next stage, fixed 45 households were randomly selected from each PSU, and within these households, all eligible respondents were identified according to the following eligibility criteria: (i) reproductive-aged ever-married women aged 15–49 years, (ii) usual residents of the selected households (who had spent the last night there). The participants provided information on various aspects of maternal and child health, including mental health conditions. Further details of sampling (Fig. 1) and the survey design are published elsewhere.^30^

For this study, we included ever-married women of reproductive age in the analytical sample, as the BDHS sampling framework designed to be restricted to this population. The other inclusion criteria for this study were: (i) who completed the mental health questionnaire, and (ii) identified as usual residents of selected households (de jure population) (Fig. 1).

The mental health module of the BDHS 2022 used two validated self-reported scales: (i) the 9-item Patient Health Questionnaire (PHQ-9); and (ii) the 7-item Generalised Anxiety Disorder scale (GAD-7). The PHQ-9 and GAD-7 assess depressive and anxiety symptoms, respectively, over the preceding two weeks using a 4-point Likert scale (0 = “not at all” to 3 = “nearly every day”), producing total scores of 0–27 and 0–21.^31^ Both scales are well-validated^32^ and widely used in epidemiological research and demonstrate very high sensitivity and specificity of approximately 80–90% for detecting depression and generalised anxiety disorder (GAD).^31^ These scales have been widely validated in similar LMIC settings as well.^32^

The primary outcomes were symptoms of depression and anxiety, considered as nominal categorical variables. Depressive symptoms were assessed using the PHQ-9,^31^ with total scores classified as no symptoms (score 0–4), mild (score 5–9), moderate to severe symptoms or probable depression (score 10–27).^31^ Anxiety symptoms were measured using the GAD-7,^31^ with total scores categorised as no symptoms (score 0–4), mild symptoms (score 5–9), moderate to severe symptoms or probable generalised anxiety disorder (GAD) (score 10–21).^31^

We considered seven HEC indicators as the primary exposures in this study to characterise the quality of the household environment. These included housing materials, household overcrowding, HAP, and WASH-related indicators.^11^^,^^12^ These indicators were selected based on their availability in the BDHS and their established relevance in characterising HEC in prior DHS-based studies from LMICs.^11^^,^^12^ Housing materials were assessed based on the construction quality of the roof, floor, and walls, with finished or improved materials in all three components considered indicative of a sound and protective dwelling.^11^^,^^12^ WASH indicators include access to safe drinking water source, water treatment, improved sanitation, and proper hygiene, reflecting the household's water, sanitation, and hygiene environment.^11^^,^^12^ We used a proxy indicator for HAP exposure defined by cooking location either indoor in a separate room (kitchen)/outdoor or inside the living area, potentially reducing indoor air quality. Household overcrowding was also included defined as more than two people share a sleeping room, reflecting stressors associated with limited living space.^11^ Details of the exposures and their categorisation are provided in Supplementary Table S1.

To assess the overall quality of the household environment, we calculated a HEC score that is considered as an additional primary exposure variable in this study. The score was derived from all seven indicators: poor housing materials, overcrowding in the household, high exposure to HAP, unimproved water source, inappropriate water treatment, and inadequate sanitation and handwashing facilities.^12^ Each poor HEC indicator was assigned a value of 1, and the individual values were aggregated to generate a composite score for each household.^12^ For example, a household with three poor HEC indicators would receive a score of 3. Higher composite scores therefore reflected poorer HECs, while lower scores indicated relatively better conditions.^12^

Other covariates included in this study were identified through literature searches and existing evidence from LMICs, including Bangladesh.^12^^,^^22^^,^^24^^,^^25^^,^^33^ Covariates were selected a priori based on their theoretical and clinical relevance as potential confounders. The covariates we included were administrative divisions (regions) of the country (Barishal, Chattogram, Dhaka, Khulna, Mymensingh, Rajshahi, Rangpur, Sylhet), place of residence (rural, urban), religion (Muslim, non-Muslim), household size (1–4 members, 5+ members), women's current age (15–19 years, 20–34 years and 35–49 years), women's education level (no education, primary, secondary or above), husband's education level (no education, primary, secondary or above), women's current working status (unemployed, employed/self-employed), duration of residence (years living in the current house) and media exposure (non-exposed, moderately exposed, and highly exposed).

### Statistical analysis

We used descriptive statistics to summarise the background characteristics, prevalence of symptoms of depression and anxiety, and the distribution of HEC indicators across the study population. Bivariate analyses were conducted to examine the distribution of HEC indicators across PHQ-9 depression and GAD-7 anxiety categories. To assess the extent to which different exposures and outcomes were associated, we applied multinomial logistic regression models, which are appropriate for nominal outcomes with more than two categories and allows direct estimation of relative risk ratios (RRs). The models were adjusted for covariates such as administrative division, place of residence, religion, household size, women's age, education, working status, husband's education, duration of residence, and media exposure. For adjusted models, RRs were estimated to quantify the strength of association, with 95% confidence intervals (95% CI) calculated for significance testing. Statistical significance was defined by a p-value threshold of <0·05. Given the cross-sectional study design, all analyses are exploratory and aimed at assessing associations rather than causal relationships. To prevent potential issues with multicollinearity, independent models were fitted for each exposure (HEC indicators), adjusting for the same set of covariates. Given the complex survey design of the BDHS, all analyses, were weighted and adjusted for clustering and stratification. All statistical analyses were performed using Stata version 18·0 MP (StataCorp LP, College Station, Texas).

### Ethics statement

This study analysed publicly available, de-identified data from the Bangladesh Demographic and Health Survey (BDHS), approved by the Government of Bangladesh. The study data were obtained from the MEASURE DHS Archive, and the authors received formal permission to use the datasets. The survey protocol of 2022 BDHS received ethical clearance from the ICF Institutional Review Board and the Bangladesh Medical Research Council (BMRC) (BMRC/NREC/2019–2022/156).

### Role of the funding source

The funding agency had no role in study design, data analysis, interpretation, or writing of the manuscript.

## Results

A total of 19,012 women were included in our analysis (Fig. 1), and the characteristics of the study participants is presented in Table 1. The majority of women resided in rural areas (71·34%), well over half (59·22%) completed secondary or higher education, and a substantial proportion (67·12%) were currently employed. Over 90% of the participants were Muslim, and half of the households (50·22%) were classified as large families in terms of household size. While 52·05% of women had moderate exposure to mass media, more than 42% reported no media exposure at all (Table 1).

**Table 1:** Characteristics of study participants from the Bangladesh Demographic and Health Survey (BDHS) 2022, (weighted n = 19,080).

Characteristics	Frequency, n	% (95% CI)
Division (Region)		
Barishal	1133	5·94 (5·62–6·27)
Chattogram	3548	18·60 (17·57–19·67)
Dhaka	4896	25·66 (24·60–26·75)
Khulna	2278	11·94 (11·39–12·5)
Mymensingh	1425	7·47 (7·16–7·79)
Rajshahi	2502	13·11 (12·36–13·90)
Rangpur	2191	11·48 (11·08–11·90)
Sylhet	1107	5·80 (5·54–6·08)
Residence		
Urban	5467	28·66 (27·50–29·84)
Rural	13,613	71·34 (70·16–72·5)
Current age		
15–19 years	1521	7·97 (7·49–8·48)
20–34 years	9619	50·42 (49·52–51·31)
35–49 years	7940	41·61 (40·71–42·52)
Level of education		
No Education	2700	14·15 (13·30–15·05)
Primary	5081	26·63 (25·75–27·53)
Secondary or above	11,299	59·22 (57·91–60·52)
Husband's education		
No Education	3994	22·06 (21–23·16)
Primary	5186	28·64 (27·64–29·66)
Secondary or above	8926	49·30 (47·82–50·79)
Current working status		
Unemployed	12,807	67·12 (65·59–68·62)
Employed/self-employed	6273	32·88 (31·38–34·41)
Religion		
Muslim	17,222	90·26 (88·27–91·94)
Other	1858	9·74 (8·06–11·73)
No of people in household		
1–4 members (low density household)	9499	49·78 (48·66–50·91)
5 or more members (medium or high-density household)	9582	50·22 (49·09–51·34)
Mass media exposure		
Non-exposed	8072	42·31 (40·71–43·93)
Moderately exposed	9932	52·05 (50·54–53·56)
Highly exposed	1076	5·64 (5·15–6·17)

Calculated for column percentages and all the analyses are weighted considering the complex survey design.

Among the weighted sample of 19,080 women, 4581 (24·06%) reported mild depressive symptoms (PHQ-9 score 5–9), while 949 (4·98%) had probable depression (PHQ-9 score ≥10). Mild anxiety (GAD-7 score 5–9) were reported by 4265 women (22·37%), and 842 (4·42%) had probable GAD (GAD-7 score ≥10). Comorbid GAD and depression were observed in 465 women (2·44%) (Table 2).

**Table 2:** Prevalence of symptoms of depression and anxiety among women from the BDHS 2022, (weighted n = 19,080).

Outcomes	Symptom score	n (%)
Symptoms of depression^a^		
No symptoms	PHQ-9 score <5	13,510 (70·96%)
Mild symptoms	PHQ-9 score 5–9	4581 (24·06%)
Moderate-to-severe symptoms (probable depression)	PHQ-9 score ≥10	949 (4·98%)
Symptoms of anxiety^b^		
No symptoms	GAD-7 score <5	13,955 (73·21%)
Mild symptoms	GAD-7 score 5–9	4265 (22·37%)
Moderate-to-severe symptoms (probable generalised anxiety disorder [GAD])	GAD-7 score ≥10	842 (4·42%)
Comorbid mental health condition^c^		
No condition	PHQ-9 & GAD-7 <9	17,712 (93·04%)
Any mental health condition	PHQ-9 or GAD-7 ≥10	861 (4·52%)
Comorbid depression and GAD	PHQ-9 & GAD-7 ≥10	465 (2·44%)

Calculated for column percentages and all the percentages are weighted to account for the survey design.

a Missing = 41.

b Missing = 18.

c Missing = 42.

Table 3 presents the distribution of HEC indicators among the study participants. Overall, more than a quarter of the respondents (4745; 28·35%) were living in households with four or more poor HEC characteristics, followed by 3958 (23·64%) were exposed to at least three, and 4017 (24·00%) were exposed to two poor HEC characteristics. Nearly half of the participants (49·35%) resided in houses constructed with poor-quality materials, and 14·14% of households were overcrowded relative to their family size. Although the majority (98·13%) had no or moderate exposure to HAP from cooking, 1·87% reported high exposure. Regarding access to drinking water, 20·17% relied on unimproved sources within their dwellings, and only 10·95% reported using appropriately treated water. In terms of sanitation, 39·07% of households had poor sanitation facilities, and 43·83% lacked proper handwashing facilities as well (Table 3).

**Table 3:** Distribution of household environmental condition (HEC) indicators among study participants (weighted n = 19,080).

Household environmental condition (HEC) indicators	Frequency, n	% (95% CI)
Housing material^a^		
Standard/improved	9436	50·65 (48·87–52·43)
Poor/unimproved	9193	49·35 (47·57–51·13)
Overcrowding		
Not overcrowded	16,382	85·86 (84·98–86·70)
Overcrowded	2698	14·14 (13·30–15·02)
Household air pollution from cooking		
Unexposed or moderately exposed	18,724	98·13 (97·71–98·48)
Highly exposed	356	1·87 (1·52–2·29)
Drinking water source in own dwelling^b^		
Improved	13,803	79·83 (77·96–81·58)
Unimproved	3486	20·17 (18·42–22·04)
Drinking water treatment		
Safe (appropriately treated)	2089	10·95 (9·57–12·50)
Unsafe (inappropriate or no treatment)	16,992	89·05 (87·50–90·43)
Sanitation facility		
Basic/standard sanitation	11,352	60·93 (59·31–62·54)
Poor sanitation	7278	39·07 (37·46–40·69)
Handwashing facilities^c^		
Standard	10,634	56·17 (54·48–57·85)
Poor	8298	43·83 (42·15–45·52)
Poor HEC score groups^d^		
≤1 poor HEC characteristics	4018	24·01 (22·50–25·58)
2 poor HEC characteristics	4017	24·00 (23·00–25·03)
3 poor HEC characteristics	3958	23·64 (22·70–24·61)
4 or more poor HEC characteristics	4745	28·35 (26·88–29·87)

Calculated for column percentages and all the percentages are weighted considering the complex survey design.

a Missing = 451.

b Missing = 1791.

c Missing = 148.

d Missing = 2341.

Bivariate analyses showed that women living in houses built with unimproved materials and in overcrowding had slightly higher prevalence of probable depression than their counterparts. The highest prevalence was observed among those exposed to high HAP (depression: 7·39% vs. 4·94%; GAD: 6·44% vs. 4·38%). Differences by drinking water source and sanitation were minimal, although slightly higher depressive symptoms were observed among those with poor sanitation and unsafe water treatment. Depression increased slightly with higher HEC scores (3·76%–4·99%), while no clear pattern was observed for GAD symptoms (Table 4).

**Table 4:** Bivariate distribution of household environmental condition (HEC) indicators across PHQ-9 depression and GAD-7 anxiety categories (weighted n = 19,080).

Household environmental condition (HEC) indicators	Symptoms of depression, n (%)		Symptoms of anxiety, n (%)	
	Mild symptoms	Probable depression	Mild symptoms	Probable GAD
Housing material				
Standard/improved	2147 (22·79%)	440 (4·67%)	1869 (19·82%)	400 (4·24%)
Poor/unimproved	2339 (25·51%)	487 (5·31%)	2305 (25·10%)	420 (4·58%)
Overcrowding				
Not overcrowded	3942 (24·11%)	799 (4·89)	3607 (22·04%)	708 (4·33%)
Overcrowded	639 (23·74%)	149 (5·54%)	658 (24·40%)	134 (4·97%)
Household air pollution from cooking				
Unexposed or moderately exposed	4474 (23· 95%)	922 (4·94%)	4167 (22·28%)	819 (4·38%)
Highly exposed	107 (30·13%)	26 (7·39%)	98 (27·47%)	23 (6·44%)
Drinking water source in own dwelling				
Improved	3315 (24·06%)	659 (4·78%)	3081 (22·33%)	571 (4·14%)
Unimproved	809 (23·29%)	172 (4·96%)	779 (22·39%)	165 (4·75%)
Drinking water treatment				
Safe (appropriately treated)	469 (22·50%)	88 (4·23%)	413 (19·82%)	111 (5·31%)
Unsafe (inappropriate or no treatment)	4112 (24·25%)	860 (5·07%)	3852 (22·69%)	732 (4·31%)
Sanitation facility				
Basic/standard sanitation	2663 (23·50%)	531 (4·68%)	2414 (21·28%)	498 (4·39%)
Poor sanitation	1823 (25·12%)	397 (5·47%)	1760 (24·21%)	322 (4·43%)
Handwashing facility				
Standard	2437 (22·97%)	511 (4·82%)	2214(20·84%)	473 (4·45%)
Poor	2120 (25·60%)	435 (5·25%)	2025 (24·43%)	367 (4·43%)
Poor HEC score groups				
≤1 poor HEC characteristics	852 (21·26%)	150 (3·76%)	724 (18·05%)	148 (3·70%)
2 poor HEC characteristics	987 (24·63%)	188 (4·68%)	892 (22·22%)	167 (4·18%)
3 poor HEC characteristics	971 (24·58%)	235 (5·95%)	937 (23·68%)	194 (4·91%)
4 or more poor HEC characteristics	1204 (25·45%)	236 (4·99%)	1194 (25·20%)	204 (4·30%)

Calculated for column percentages and all the percentages are weighted considering the complex survey design.

The association of HEC indicators with the symptoms of anxiety and depression among women is presented in Table 5. High cumulative poor HEC score was associated with symptoms of depression, but not with GAD. Women residing in households with three (aRR: 1·30, 95% CI: 1·13–1·83) and four or more poor HEC characteristics (aRR: 1·31, 95% CI: 1·02–1·67) had a higher likelihood of probable depression, compared to those with one or fewer HECs, after adjusting for covariates such as regions, place of residence, religion, household size, women's age, working status, education, husband's education, duration of residence, and media exposure.

**Table 5:** Association of household environmental condition (HEC) with symptoms of depression and anxiety in the BDHS 2022 (weighted n = 19,080).

Household environmental condition (HEC) indicators	Symptoms of depression, Adj. RR (95% CI)		Symptoms of anxiety, Adj. RR (95% CI)	
	Mild symptoms	Probable depression	Mild symptoms	Probable GAD
Housing materials				
Standard/improved (ref)	1·00	1·00	1·00	1·00
Poor/unimproved	1·09 (1·00–1·19)	1·02 (0·86–1·20)	1·17 (1·08–1·28)	1·00 (0·84–1·20)
Overcrowding				
Not overcrowded (ref)	1·00	1·00	1·00	1·00
Overcrowded	1·04 (0·94–1·16)	1·20 (0·98–1·46)	1·18 (1·06–1·30)	1·32 (1·07–1·63)
Household air pollution from cooking				
Unexposed or moderately exposed (ref)	1·00	1·00	1·00	1·00
Highly Exposed	1·51 (1·18–1·93)	1·58 (1·01–2·45)	1·28 (0·99–1·65)	1·61 (1·02–2·57)
Drinking water source in own dwelling				
Improved (ref)	1·00	1·00	1·00	1·00
Unimproved	1·13 (0·96–1·33)	1·11 (0·92–1·34)	0·94 (0·85–1·04)	1·11 (0·90–1·36)
Drinking water treatment				
Safe (appropriately treated) (ref)	1·00	1·00	1·00	1·00
Unsafe (inappropriate or no treatment)	1·14 (0·97–1·33)	1·17 (0·83–1·64)	0·92 (0·78–1·09)	0·61 (0·45–0·83)
Sanitation facility				
Standard/basic sanitation (ref)	1·00	1·00	1·00	1·00
Poor sanitation	1·13 (1·05–1·22)	1·21 (1·05–1·39)	1·18 (1·10–1·28)	1·07 (0·92–1·25)
Handwashing facility				
Standard (ref)	1·00	1·00	1·00	1·00
Poor	1·16 (1·07–1·25)	1·07 (0·92–1·25)	1·14 (1·05–1·23)	0·97 (0·83–1·14)
Poor HEC score groups				
≤1 poor HEC characteristics (ref)	1·00	1·00	1·00	1·00
2 poor HEC characteristics	1·19 (1·07–1·33)	1·18 (0·93–1·50)	1·18 (1·05–1·33)	1·05 (0·82–1·34)
3 poor HEC characteristics	1·21 (1·08–1·36)	1·30 (1·13–1·83)	1·27 (1·12–1·43)	1·16 (0·90–1·50)
4 or more poor HEC characteristics	1·26 (1·12–1·42)	1·31 (1·02–1·67)	1·36 (1·21–1·54)	1·08 (0·83–1·39)

Values in “No Anxiety symptoms” and “No depressive symptoms” are considered as reference value. Separate models were fitted for each exposure, adjusted for division, residence, women's current age, women's education, women's current working status, religion, no. of people in household, husband's education, mass media exposure and duration of living in the household. (ref): Reference category, RR: risk ratio, CI: confidence interval.

Poor HEC scores showed a dose-response relationship with mild symptoms of depression or anxiety. The risk of mild depression symptoms was increased by 1·19 times (95% CI: 1·07–1·33), 1·21 times (95% CI: 1·08–1·36), and 1·26 times (95% CI: 1·12–1·42) and the risk of mild anxiety was increased by 1·18 times (95% CI: 1·05–1·33), 1·27 times (95% CI: 1·12–1·43), and 1·36 times (95% CI: 1·21–1·54) among women residing in households with two, three, and four or more poor HEC characteristics, respectively, compared to their counterparts.

With regards to individual HEC indicators, exposure to high levels of HAP was associated with both probable depression (aRR: 1·58, 95% CI: 1·01–2·45) and GAD (aRR: 1·61, 95% CI: 1·02–2·57). Overcrowding was associated with GAD (aRR: 1·32; 95% CI: 1·07–1·63). Poor sanitation was associated with depression (aRR: 1·21, 95% CI: 1·05–1·39) and mild anxiety (aRR: 1·18, 95% CI: 1·10–1·28). Poor access to handwashing facility at home was associated with mild anxiety (aRR: 1·14, 95% CI: 1·05–1·23) and mild depression (aRR: 1·16, 95% CI: 1·07–1·25) (Table 5).

## Discussion

This study evaluates the association between HECs and common mental health outcomes among women in Bangladesh. We found that 5·0% and 4·42% of women had probable depression and GAD, respectively, while 2·44% experienced both. HECs were found generally poor, with over half of households with three or more poor characteristics. HEC indicators, for example, high exposure to HAP was significantly associated with both depression and GAD, whereas overcrowding was linked to GAD and poor sanitation facilities increased the risk of depression. In contrast, poor housing materials and lack of proper handwashing facilities were associated only with mild depression and anxiety. We found that higher cumulative poor HEC scores were associated with clinically relevant depression but not GAD. Dose–response patterns of these associations were observed only for mild symptoms.

Our estimated prevalence of clinically relevant depression symptoms in a large sample of women in Bangladesh is consistent with prior national estimates (∼5% among women aged 15–49),^22^^,^^24^^,^^25^ though slightly lower than the 6·7% reported in the Bangladesh National Mental Health Survey (NMHSB) (2018–19) among adults in general. However, our findings on GAD are similarly comparable with NMHSB 2018–19 estimate (4·5% adults)^34^; in contrast, some previous studies reported a 4–5 times higher prevalence than our estimate due to the use of a lower, less clinically relevant threshold of GAD-7.^24^^,^^25^ A recent nationally representative study reported noticeably higher depression prevalence (∼16%), likely due to sampling of socio-environmentally vulnerable households and repeated surveys conducted during climate-related stress, capturing transient rather than persistent distress.^32^ Compared with the global estimates, the prevalence of depression and GAD was slightly lower than that reported in the Global Burden of Disease 2021 study and in high income countries.^1^^,^^2^ These differences may reflect stigma, cultural expressions of distress, and healthcare access, which may influence reporting patterns.^35^ Nevertheless, we still confirm a substantial burden of depression and anxiety in reproductive age women in Bangladesh, which in absolute terms represent an estimated ∼2·1–2·4 million people.

Several indicators of HEC have increasingly been recognised as a risk factors of common mental disorders (CMDs) in the global literature. Firstly, our findings, which identify high HAP exposure from cooking as a key risk factor of depression and GAD among women, aligns well with findings from diverse settings.^16^^,^^36^^,^^37^ However, research specifically addressing women's exposure to cooking-related HAP remains more limited compared with studies on second-hand smoke.^16^^,^^36^^,^^37^ Recent reviews further confirm that HAP arising from solid fuel use for cooking or heating, as well as second-hand smoke, increases the risk of depression.^16^^,^^37^ On the other hand, although we observed a relationship between household overcrowding and GAD, direct global evidence supporting this relationship remains limited. An adolescent-based study concluded that overcrowding increases anxiety only when it combines with poverty or other housing-related stressors.^38^ Rather than GAD, its relationship with depression is more evident in the existing literature^17^^,^^39^; however, our analysis did not observe such association. The World Health Organization highlights household overcrowding as a risk factor of CMDs in general,^40^ but very limited studies established this relationship in LMIC context.

Our findings regarding sanitation also consistent with global evidence that concludes poor sanitation increases the risk of depression.^15^^,^^33^ A recent meta-analysis covering 16 countries further give more strengths to these relationships with CMDs.^15^ However, most prior studies have focused largely on general populations, and our study underscores the heightened vulnerability of reproductive-aged women in LMICs, who are disproportionately exposed to household-level risks. We also observed, women living in houses built with unimproved materials or lacking proper handwashing facilities had higher risks of mild symptoms, but not GAD or depression. This partially contrasts with evidence that living long-term in poor-quality housing adversely affects mental health beyond immediate housing stressors,^41^ which we could not assess due to the cross-sectional nature of our data. Research directly linking mental health to handwashing facilities is very limited, instead most existing studies focuses on handwashing behaviours, which linked to the access, showing varied or inconsistent associations.^42^ We observed no clear link between drinking water source and mental health, a findings that contrasts with evidence more commonly reported in Sub-Saharan Africa.^15^

However, HECs interact with mental health conditions in distinct pathways comprising both biological and psychosocial factors. For example, HAP produces fine particulate matter (PM_2·5_, PM_10_) that triggers neuro-inflammation and oxidative stress that significantly increases depression risk.^43^^,^^44^ This exposure also activates systemic inflammation and dysregulates the hypothalamic–pituitary–adrenal (HPA) axis,^43^^,^^44^ which is body's central stress response system. Similarly, poor sanitation and hygiene increase pathogen exposure that activate systemic inflammation as well.^45^ Besides, overcrowding and living in houses built with poor materials create chronic stress.^17^^,^^40^^,^^41^ This persistent stress dysregulates the HPA axis, and elevates cortisol levels as well. Additionally, the physical discomfort from poor housing, including leaky roofs, inadequate insulation, damn condition, and poor ventilation, contributes to disrupted sleep^46^ and thermal stress.^47^ Thus, all these poor HEC characteristics collectively disrupt neurotransmitter function and stress regulation that provides a plausible biological link to depression and GAD among women.^43^^,^^44^ Beyond biological mechanisms, women in LMICs, like in Bangladesh, particularly in lower socioeconomic groups, spend most of their time indoors and bear primary responsibility for cooking and household chores.^14^ This longer daily indoor exposure, combined with sex-specific vulnerabilities and domestic roles, compounds women's risk of poor mental health. Furthermore, poor sanitation, housing and overcrowding exacerbate chronic psychological stress by reducing privacy, causing discomfort, increasing fear of violence, noise, and interpersonal conflict.^48^^,^^49^ Over time, these stressors may accumulate into chronic strain, increasing the risk of anxiety and depression. Additionally, poor sleep quality, common in overcrowded homes,^50^ further elevates the risks.^51^ These overlapping psychosocial, biological, and caregiving stressors likely interact altogether to amplify the burden of depression and GAD in Bangladesh.

The study also indicated a strong aggregated association between poor HEC scores and the likelihood of depression. While each HEC indicators independently contribute to depression or GAD through their biopsychosocial pathways, their cumulative effect is expected to be amplified further, although we did not find such association for GAD. For instance, a women living in a household with high exposure to HAP might already face an increased risk of depression due to the potential exposure to particulate matters. If that household also lacks improved sanitation facilities, or having any other poor characteristics, the combined impact of all these factors likely compounds the psychological impact beyond that of any single individual HEC factors. Future research should explore both individual and cumulative HEC pathways using longitudinal designs to better establish temporality and examine mechanisms underlying the combined effects of multiple household environmental exposures on mental health outcomes.

There are several strengths and limitations that should be taken into consideration when interpreting the results. Firstly, we used a large nationally representative data and further, we used a multinomial regression model to explain the relationship, which effectively accounts for potential effects related to mild anxiety and depression, thus, enhancing the robustness of our findings. This study also included direct measures of daily living environments, going beyond broader SES, which contributes to a more comprehensive understanding of the daily-life context in which mental health outcomes occur. Lastly, the composite scoring of HECs has provided important insights into the associations being explored. However, the study also has several limitations. Depression and GAD were assessed using screening instruments (PHQ-9 and GAD-7), which capture current symptoms rather than clinical diagnoses and may be subject to reporting bias. However, both instruments have high sensitivity and specificity and are widely used in population studies. We analysed data of ever-married women only, which restricted our ability to provide estimates for unmarried women or women living in other household arrangements. Moreover, the cross-sectional design of the survey restricts causal inference. Reliance on self-reported measures may introduce recall and social desirability biases, and that could affect the accuracy of results. Certain HEC variables, including household overcrowding or water treatment, were measured crudely, possibly leading to misclassification. The BDHS 2022 wave also lacked data on types of cooking fuel used that limit our ability to distinguish moderate exposure group. Although missing data were minimal, some covariates and exposures had incomplete observations, which may introduce residual bias. Unmeasured confounding from socioeconomic or cultural factors may further influence observed associations. Finally, while the HEC composite captured multiple dimensions of housing quality, it did not include factors such as ventilation, insulation, adequacy of space, heating or cooling facilities.

In conclusion. This study provides evidence linking HECs to women's mental health in Bangladesh. We found that depression and GAD affect approximately one in twenty women, and the environmental conditions of most of the households where the women live were generally poor. Several HEC indicators, including high HAP exposure, inadequate sanitation, and overcrowding, increased the likelihood of adverse mental health conditions among women, even after accounting for traditional SES markers. A cumulative burden of poor HECs had a stronger relationship with depression, suggesting that co-occurring HEC exposures may compound mental health risk further. These findings suggest that HECs could be potentially modifiable risk factors of women's mental illness and that community interventions such as reducing exposure to HAP, promoting improved sanitation and addressing overcrowding in the household may help improving women's mental health. Further research is required to replicate these findings in other populations, and to examine potential mechanisms and evidence of causality.

## Contributors

MMAK conceptualised and designed the study. KF conducted the literature review. MMAK, KF, and MNIS carried out data management and statistical analyses. MMAK, KF, MNIS, MAB, MGM, and SR contributed to drafting the initial version of the manuscript. MN Khan and GMK provided critical review and substantive revisions of earlier manuscript drafts. GMK supervised the entire study. MMAK, KF, and MAB had full access to and verified the underlying data. All authors approved the final version of the manuscript.

## Data sharing statement

This study used publicly available data of Demographic and Health Survey programme. The dataset can download after registering with the MEASURE DHS at: https://dhsprogram.com/data/available-datasets.cfm.

## Declaration of interests

GMK has received royalties from the Cambridge University Press for the Textbook of Immunopsychiatry, and consultancy and speaker fee from the Neuroimmune Foundation and the Danish Research Fund. The authors have no other interest to declare.

## Appendix A Supplementary data

Supplementary Table

Ethical Review
