# Supplementary material for: Association between household environment and the risk of depression and anxiety among women in Bangladesh: analysis of the Bangladesh Demographic and Health Survey (2022)
Source: Lancet Reg Health Southeast Asia. 2026 Aug 17;52:100840. doi: 10.1016/j.lansea.2026.100840 (PMC13505358; doi:10.1016/j.lansea.2026.100840)
Supplement: Supplementary file 1 [file mmc2.pdf]

Subject: Re: New ethical review documentation request
Date: Tuesday, 3 February 2026 at 16:13:18 Greenwich Mean Time
From: Hanner, Olsen
To: mostaured.khan@bristol.ac.uk
CC: irbrequest@dhsprogram.com
Attachments: Approval_Ethical (BMRC)_BDHS-2021-2022_9June2022.pdf, ICF IRB Approval .pdf

Hello Md Mostaured Ali Khan,

Please find attached the ICF and local ethical review documentation for the 2022 Bangladesh DHS. The study was determined to be 'NHSR - Not Human Subjects Research.'

While neither of these attached documents explicitly states that analysis using these datasets does not require any further IRB approval, your authorization letter does outline that DHS Program public-use datasets have undergone IRB-approved procedures before being released. You do not need further IRB approval to use the publicly distributed datasets, as long as you abide by the Terms of Use outlined in the letter.

Sincerely,
Olsen

Olsen Hanner
The Demographic and Health Surveys (DHS) Program
Data Archivist | +1.301.407.6627 | olsen.hanner@icf.com
ICF | 530 Gaither Road, Suite 500, Rockville MD 20850 USA

Access data: Dataset Archives | STATcompiler | Mobile App | Spatial Data Repository | API
Get help: Learning Hub | YouTube | User Forum | Code Share Library

---

From: irbrequest@dhsprogram.com <irbrequest@dhsprogram.com>
Sent: Tuesday, February 3, 2026 7:45 AM
To: irbrequest@dhsprogram.com <irbrequest@dhsprogram.com>
Subject: New ethical review documentation request

A new ethical review documentation request has been received:

Name: Md Mostaured Ali Khan
Email: mostaured.khan@bristol.ac.uk
Survey(s): Bangladesh Demographic and Health Survey 2022
Reason: We have submitted a research paper to Lancet Regional Health - Southeast Asia, based on this dataset BDHS 2022. While I have the data access approval letter, the editors have requested further documentation regarding the original ethical approval (with IRB name and number) of the survey collection to satisfy their transparency requirements. Could you provide any standard document or formal statement regarding the IRB/Ethics Committee

approval that covered the 2022 BDHS data collection? I have provided them the IRB name but they are still requesting for further information. They want us to provide a document that proved the analysis using this dataset does not required any IRB further (waived). Looking forward to hearing back from you. We would be so grateful if you provide help us in this situation. Regards Mostaured Khan
Journal: Lancet Regional Health - Southeast Asia

বাংলাদেশ চিকিৎসা গবেষণা পরিষদ
Bangladesh Medical Research Council
BMRC

Ref: BMRC/NREC/2019-2022/156 Date: 06/06/2022

Mr. S N Mitra
Executive Director
Mitra and Associates
Commercial Plot # 35 (3rd – 5th Floor)
Main Road # 01, Section # 10
Senpara Porbota, Mirpur, Dhaka-1216

Subject: Regarding renewal of the project.

Research proposal entitled **"Bangladesh Demographic and Health Survey (BDHS) 2021 – 2022"** was submitted on 01st June, 2022 for renewal of the proposal.

As there is no change in the previous protocol submitted in 2017, renewal has been approved by the National Research Ethics Committee.

Thanking you,

6/6/22

(Prof. Dr. Md. Ruhul Amin)
Director

BMRC Bhaban, Mohakhali, Dhaka-1212, Bangladesh.
Phone: +88 02 9848396, PABX : +88 02 9849311, Fax : +88 02 9848820, E-mail: info@bmrcbd.org. Web: www.bmrcbd.org
