# Supplementary material for: Association between household environment and the risk of depression and anxiety among women in Bangladesh: analysis of the Bangladesh Demographic and Health Survey (2022)
Source: Lancet Reg Health Southeast Asia. 2026 Aug 17;52:100840. doi: 10.1016/j.lansea.2026.100840 (PMC13505358; doi:10.1016/j.lansea.2026.100840)
Supplement: Supplementary file 2 [file mmc1.docx]

**Supplementary table 1.** Operational definition of the variables of interest

| **Outcome** | **Definition** | **Categories** |
| --- | --- | --- |
| Anxiety symptoms | Anxiety symptoms were assessed using the 7-item Generalised Anxiety Disorder scale (GAD-7). Respondents reported the frequency of seven anxiety-related problems experienced during the past two weeks on a 4-point Likert scale (0 = “not at all”, 1 = “several days”, 2 = “more than half the days”, 3 = “nearly every day”). The total score ranges from 0–21. The GAD-7 has been validated in multiple populations and shows high sensitivity (89%) and specificity (82%) for detecting generalised anxiety disorder (GAD) ^1^. | 1 = No symptoms (0–4)  2 = Mild symptoms (5–9)  3 = Moderate to severe symptoms (10–21)/ **Presence of GAD**: Score ≥10 |
| Depressive symptoms | Depressive symptoms were measured using the 9-item Patient Health Questionnaire (PHQ-9). Respondents indicated the frequency of nine depressive symptoms over the preceding two weeks on the same 4-point Likert scale as the GAD-7 (0–3). The total score ranges from 0–27. The PHQ-9 is widely used in clinical and population-based studies, with high sensitivity (80–90%) and specificity (90%+) for detecting depression ^1^. | 1 = No symptoms (0–4)  2 = Mild symptoms (5–9)  3 = Moderate to severe symptoms (10–27)/ **Presence of depression**: Score ≥10 |
| Comorbid mental health condition | A comorbid mental health condition was defined as the co-occurrence of both anxiety and depressive symptoms, defined as respondents scoring ≥10 on both the GAD-7 and the PHQ-9. This classification identifies individuals experiencing overlapping symptoms of depression and GAD, who are considered at particularly high risk for adverse outcomes. | 0 = No symptoms  1 = Any mental health condition  2 = Comorbid depression and GAD |
| **Household environment condition (HEC) indicators^2,3^** | | |
| Housing materials | The household that built with finished roof, floor and wall materials are improved household. Finished floor materials are parquet or polished wood, ceramic tiles, cement, carpet; finished wall materials are cement, stone with lime/cement, and cement blocks; and finished roof materials are metal, calamine, cement fibre, ceramic tiles, cement and roofing shingles.  Unimproved material refers to the use of natural or rudimentary materials like earth/sand, dung, wood plunks, palm/bamboo, cardboard, rustic mat, etc. | 1 = Standard/improved  2 = Poor/unimproved |
| Overcrowding | Overcrowding in the household was assessed using the number of usual household members per sleeping room. Overcrowding occurs when more than two people share a single room used for sleeping. | 1 = Not overcrowded  2 = Overcrowded (>2 persons per sleeping room) |
| Household air pollution (HAP) from cooking | In earlier BDHS surveys, HAP exposure was defined based on the type of cooking fuel used (clean or solid fuels) and the location of cooking. Respondents were classified as no- to low-exposure if they either used clean fuels or used solid fuels in a separate building or outdoors, and as high exposure if solid fuels were used for cooking inside the house. For the BDHS 2022 dataset, information on fuel type was not collected. Therefore, in this study we used a proxy measure of HAP exposure derived only from cooking location. Households where cooking was conducted indoors in a separate kitchen or outdoors were considered low exposure, while those cooking inside the main living area were categorised as high exposure, reflecting poorer indoor air quality. This categorisation follows the approach adopted in previous LMIC studies. | 0 = Unexposed/Moderately exposed  1 = Highly exposed |
| Drinking water source in own dwelling | The sources of drinking water in the own dwelling are collected from any unprotected well/borehole/tube well, river/dam/lake/ponds/stream/canal and cart with small tank are considered as the unimproved water source, otherwise the water collected from piped facility, tube well or borehole, protected well, rainwater, tanker truck or bottled water are considered as improved water source. | 1 = Improved water source  2 = Unimproved water source |
| Drinking water treatment | The drinking water was treated for making it safe-drinking water includes boiling, bleaching or mixing chlorine, using water filter are considered as appropriately treated. | 1 = Appropriately treated  2 = Inappropriately treated/not treated. |
| Sanitation facility | Basic sanitation is defined as use of improved toilet facilities that are not shared with other households. Includes safely managed sanitation service, which is not shown separately. Improved sanitation was defined as access to a flush/pour-flush toilet connected to a sewer system, septic tank, or pit latrine, a ventilated improved pit latrine, or a pit latrine with a slab, consistent with BDHS/WHO-JMP classifications. | 1 = Basic/Standard/improved sanitation  2 = poor sanitation or open defecation |
| Handwashing facility | The household had observable handwashing place with available water and cleaning reagent (i.e., soap) are considered as the standard handwashing facility. | 1 = Standard handwashing facility  2 = Poor handwashing facility |
| **Poor household environment condition (HEC) score** | Poor household environment condition scoring is an index of the quality of the house where the respective households have one or more poor household environment quality characteristics. The score is calculated based on poor household environment quality characteristics that includes unimproved housing materials, moderate or highly IAP exposure, unimproved water source, inappropriate or no treatment of drinking water, poor sanitation facility or open defecation, poor hand-washing facilities. | 1 = ≤1 poor HEC characteristics  2 = 2 poor HEC characteristics  3 = 3 poor HEC characteristics  4 = 4 or more poor HEC characteristics |

**Reference**

1.	Kroenke K, Spitzer RL, Williams JBW, Löwe B. The Patient Health Questionnaire Somatic, Anxiety, and Depressive Symptom Scales: a systematic review. *General Hospital Psychiatry* 2010; **32**(4): 345–59.

2.	Brown C, Ravallion M, van de Walle D. Child health and the housing environment. *World Development* 2023; **168**: 106265.

3.	Khan MMA, Billah MA, Fatima K, et al. Child undernutrition and its association with household environmental conditions in Bangladesh. *Public Health Nutrition* 2025; **28**(1): e3.
